# Manipulating attentional load in sequence learning through random number generation

**DOI:** 10.2478/v10053-008-0114-0

**Published:** 2012-05-21

**Authors:** Michał Wierzchoń, Vinciane Gaillard, Dariusz Asanowicz, Axel Cleeremans

**Affiliations:** 1 Institute of Psychology, Jagiellonian University, Krakow, Poland; 2 Consciousness, Cognition, and Computation Group, Université Libre de Bruxelles, Belgium

**Keywords:** implicit learning, attention, serial reaction time task, random number generation task, tone counting task

## Abstract

Implicit learning is often assumed to be an effortless process. However, some artificial grammar learning and sequence learning studies using dual tasks seem to suggest that attention is essential for implicit learning to occur. This discrepancy probably results from the specific type of secondary task that is used. Different secondary tasks may engage attentional resources differently and therefore may bias performance on the primary task in different ways. Here, we used a random number generation (RNG) task, which may allow for a closer monitoring of a participant’s engagement in a secondary task than the popular secondary task in sequence learning studies: tone counting (TC). In the first two experiments, we investigated the interference associated with performing RNG concurrently with a serial reaction time (SRT) task. In a third experiment, we compared the effects of RNG and TC. In all three experiments, we directly evaluated participants’ knowledge of the sequence with a subsequent sequence generation task. Sequence learning was consistently observed in all experiments, but was impaired under dual-task conditions. Most importantly, our data suggest that RNG is more demanding and impairs learning to a greater extent than TC. Nevertheless, we failed to observe effects of the secondary task in subsequent sequence generation. Our studies indicate that RNG is a promising task to explore the involvement of attention in the SRT task.

## INTRODUCTION

The goal of this paper is to explore the extent to which implicit learning, the process whereby one can become sensitive to regularities contained in stimulus material in the absence of awareness, can take place under attentional load. This issue is central to any theory that depicts implicit learning as an effortless, automatic, and mandatory process that accompanies information processing (for a review, see Frensch & Rünger, 2003; Shanks, Rowland, & Ranger, 2005). Some researchers have suggested that implicit learning does indeed take place independently of the level of attention or cognitive effort (e.g., Frensch & Miner, 1994; for *automatic learning hypothesis*, see Schumacher & Schwarb, 2009). However, while there has been some convincing evidence that implicit learning may take place automatically in artificial grammar learning situations (e.g., Dienes & Scott, 2005; Hayes, 1989), not all studies have confirmed this observation (e.g., Dienes, Broadbent, & Berry, 1991). Furthermore, many studies dedicated to sequence learning have suggested that attention is in fact necessary for learning to occur (e.g., Jiménez & Vázquez, 2005; Shanks et al., 2005).

To explore the putative automatic character of implicit learning, most studies have relied on asking participants to perform a concurrent secondary task. One challenge in this respect is that different studies have often relied on different secondary tasks. Further, few studies have explored secondary task performance, which results in a limited ability to draw inferences about the effects of such tasks on the primary task. Here, we explore a novel secondary task, random number generation (RNG), which we think makes it possible both to better control participants’ level of engagement with a secondary task and to better assess performance parametrically.

In the following paragraphs, we first briefly introduce the two paradigms with which we will be concerned in this study: artificial grammar learning and sequence learning. Next, we briefly review the main findings obtained from each paradigm with respect to the effects of a secondary task on implicit learning. Finally, we introduce our own experiments.

### Implicit leaning paradigms: Similarities and differences

Artificial grammar learning, developed by Reber (1967), and sequence learning, first introduced by Nissen and Bullemer (1987), are the two main paradigms through which implicit learning has been documented. In artificial grammar learning, participants are asked to memorize meaningless letter strings that have been constructed based on a finite-state grammar. Afterwards, they are informed that all of the strings were constructed according to rules that determine the order of letter presentation. In a second phase of the task, participants classify new strings by deciding whether a given string follows the rule or not. Typical results show that although participants exhibit almost no verbal knowledge about the structure of the material, the classification performance is above chance level (Reber, 1989). In a typical sequence learning experiment, participants first perform a serial reaction time (SRT) task (Nissen & Bullemer, 1987), in which they are asked to react to each element of a sequentially structured (and most commonly visual) sequence of events. In each trial, a stimulus appears at one of several locations on a computer screen and participants are asked to press a corresponding key as fast and as accurately as possible. Unbeknownst to the participants, the sequence of successive stimuli follows a repetitive pattern. Reaction times (RTs) tend to decrease progressively during practice, but increase dramatically when the repetitive pattern is modified in any of several ways (e.g., Destrebecqz & Cleeremans, 2001). This pattern of results suggests that participants have become sensitive to the sequential regularities contained in the material during the course of training.

A common factor in both tasks is that participants acquire some information about the underlying structure of the material without having any intention of doing so. Both paradigms share similar learning conditions, that is, participants know nothing about the existence of hidden rules but behave as if they had acquired some knowledge of them. It is assumed that an implicit learning mechanism is responsible for both types of results. However, whether the nature of the knowledge acquired in both tasks is the same is still debatable (Perruchet, 2008). Most importantly, it has been shown that procedural learning of motor reactions may influence knowledge acquisition in sequence learning (Woltz, Gardner, & Bell, 2000) but may not in artificial grammar learning.

### Implicit learning and dual tasks

*Implicit learning* is usually described as unconscious, unintentional, and automatic (Jiménez & Méndez, 1999; Pothos, 2007). Thus, by definition, implicit learning should not require general processing resourcesand attentional control (e.g., Berry & Dienes, 1993; Hsiao & Reber, 1998; Jiménez & Vázquez, 2005; Shanks, 2003), and should be characterized by low vulnerability to secondary task influence (e.g., Dienes & Berry, 1997; Frensch & Miner, 1994; Reber, 1992). Experimental results have often confirmed this assumption, but the question of the role of attention in implicit learning continues to elicit debate. A number of studies have used either artificial grammar learning or SRT paradigms to investigate the effects of attentional load on implicit learning.

Studies using artificial grammar learning paradigm have shown that the requirement to perform a secondary task that demands attention does not interfere with learning (Broadbent, 1989; Dienes & Scott, 2005; Hayes, 1989) and that sometimes it even facilitates the acquisition of implicit knowledge (Perruchet, 2008). Similar results were obtained in some studies using SRT tasks that showed implicit sequence learning was observed under dual-task conditions (Cohen, Ivry, & Keele, 1990; Frensch, Buchner, & Lin, 1994; Reed & Johnson, 1994; Shanks & Johnstone, 1998). Naturally, the observation of learning under dual-task conditions does not imply that implicit learning is entirely independent of attentional resources. It is possible that the secondary task does not completely deplete attentional resources (Hsiao & Reber, 1998; Stadler, 1995). Nevertheless, the results of the experiments cited above suggest that attentional requirements for implicit learning are still lower than they are for explicit learning, which, by definition, is effortful.

Other recent studies have challenged the aforementioned assumptions by demonstrating substantial effects of attentional load in implicit learning situations. Dienes et al. (1991), and Chang and Knowlton (2004) showed that implicit learning is impaired under dual-task conditions in artificial grammar learning paradigm. However, SRT data from sequence learning paradigm are less clear-cut. Many researchers have reported that situations in which participants perform a secondary task during learning result in reduced learning and longer RTs in general (Jiménez & Vázquez, 2005; Shanks & Channon, 2002; Shanks et al., 2005; cf. Table 1.). Such results could lead us to question the extent to which implicit learning is automatic, but it is also possible that these discrepancies are caused by differences among the procedures. In either case, if one assumes that artificial grammar learning and sequence learning measure the same process, one would expect similar effects from the requirement to perform a concurrent secondary task. The results presented above suggest that this is not the case. In the next section, we analyze the possible causes of such incongruent results.

**Table 1.** A Comparison of the Use of Secondary Tasks in Artificial Grammar Learning and Sequence Learning Studies

ARTIFICIAL GRAMMAR LEARNING STUDIES			
Author(s)	Secondary task	IL under dual task	Results
Hayes (1989)	RNG	Observed	Impaired classification under intentional learning instructions (EL measure) but intact under standard incidental memory instruction (IL measure).
Dienes, Broadbent, and Berry (1991, Experiment 2)	RNG	Impaired	Impaired performance in classification and other measures of IL (d’ and sequential letter dependencies tests) both under intentional and incidental instructions.
Chang and Knowlton (2004, Experiment 2)	Articulatory suppression	Observed	Knowledge about abstract rules can be acquired but articulatory suppression reduces later sensitivity to chunk strength.
Dienes and Scott (2005)	RNG	Observed	No effects on classification performance and measures of the conscious or unconscious status of judgment knowledge (i.e., guessing criterion and Chun-difference score); decreased proportion of attributions to conscious structural knowledge (EL).	
SL STUDIES/ SRT PARADIGM			
Nissen and Bullemer (1987)	TC	Impaired	Acquisition of the sequence under TC was minimal.
Cohen, Ivry, and Keele (1990)	TC	Observed/impaired	Simple structured sequences can be learned with TC but more complex ones require attention.
Curran and Keele (1993)	TC	Observed	No influence of TC regardless of the level of sequence awareness.
Reed and Johnson (1994)	TC	Observed	SOC can be learned under TC; attention was not manipulated (no control conditions TC used to minimize opportunities for explicit learning).
Frensch, Buchner, and Lin (1994)	TC	Observed	Both unique and ambiguous sequences can be learned under TC; time of secondary task onset and time interval between the response to a stimulus and the presentation of the next stimulus affect SRT performance.
Stadler (1995)	TC, memory-load task	Observed/impaired	No influence of memory load but impaired SL under TC; TC disrupts learning by preventing consistent organization of the sequence.
Heuer and Schmidtke (1996)	Verbal, visuo-spatial, and auditory go/no-go (similar to TC) tasks	Observed/impaired	Only auditory go/no-go task (TC with no requirement of updatingand memorizing the number counted) interferes with the SL; interference seems to be specific to certain secondary tasks.
Mayr (1996)	TC, learning of a second sequence	Observed	Learning of spatial and object sequences simultaneously was as efficient as learning of single sequences; the effect occurs even under TC.
Schmidtke and Heuer (1997)	Go/no go task as in Heuer and Schmidtke (1996)	Observed/impaired	Performance decrement under dual-task conditions can be caused by a task integration that impairs SRT (reduced SRT under go/no go task with random sequences of tones; repeated sequences of tones integrated with SRT enhanced learning).
Frensch, Lin, and Buchner (1998)	TC	Impaired	TC primarily affects expression of learning (practice effects in SRT did not differ under TC), but also implicit learning itself (when learning assessment was performed under TC).
Shanks and Johnstone (1998)	TC	Observed	Replication of results reported by Reed and Johnson (1994): SOC sequences can be learned under TC.
SL STUDIES/ SRT PARADIGM			
Schvaneveldt and Gomez (1998)	TC	Observed	Probabilistic sequences (first- and second-order conditional) are learned under TC; transfer effect results under TC suggest limitations in performance but not in learning.
Jiménez and Méndez (1999)	VSC	Observed	No effect of VSC (target shape-counting performed on stimulus on which SRT was being carried out) on SL of probabilistic sequences generated with finite-state grammar.
Rah, Reber, and Hsiao (2000)	TC	Observed/impaired	Contingency of tone sequence in TC and SRT influence learning; SOC sequences can be learned under TC contingent with SRT (reverse results in Experiment 4 when TC was not contingent); attention was not manipulated across conditions.	
Jiménez and Méndez (2001)	VSC	Observed	Replication of results reported by Jiménez and Méndez (1999): SLcan be acquired and expressed under VSC even when participantscannot anticipate the next location in cued generation task (EL test).
Hsiao and Reber (2001)	TC	Impaired	Significant learning of the SOC sequence; the effect was influenced by response-secondary SOA of tones and level of TC performance.
Shanks and Channon (2002)	TC	Impaired	SL of SOC affected by TC during training regardless of the presence of TC at the transfer block.
Jiménez and Vázquez (2005)	TC, TC associated with SRT	Observed/impaired	TC affected expression and acquisition of SL; greater interference was observed with deterministic sequence (EL); no influence of TC on SL when task is associated with SRT.
Shanks, Rowland, and Ranger (2005)	VSC	Impaired	VSC impairs SL of SOC (regardless of the presence of secondary task at transfer); acquired knowledge, as assessed by generation task, was consciously accessible.
Poldrack et al. (2005)	TC	Observed	fMRI study; behavioral data: no effects of TC after intensive training; fMRI data: before training, SRT with TC elicited activation in a wide network of frontal and striatal regions as well as parietal lobe; after training, SRT under TC showed less activity in bilateral ventral premotor regions, right middle frontal gyrus, and right caudate body.
Nejati, Farshi, Ashayeri, and Aghdasi (2008)	TC	Observed/impaired	SL under TC observed in younger adults but impaired in elderly group.
Cohen and Poldrack (2008)	Letter counting task	Impaired	Letter counting task impaired SRT but dual-task effect decreasedwith training (SRT lasted 3hr).
Schumacher and Schwarb (2009)	Tone-identification task	Observed/impaired	Dual-task disrupts SRT only when the processing for the two tasks overlap(i.e., parallel response selection for both tasks interfere short SOA) and with equal priority of tasks (as compared to SRT priority).
Hemond, Brown, and Robertson (2010)	VSC, learning of a second sequence	Observed/impaired	SL can be enhanced by concurrently learning sequence of colored cues and impaired by VSC (counting the number of red cues).

*Note*. EL = explicit learning. IL = implicit learning. RNG = random number generation task. SL = sequence learning. SOA = stimulus-onset asynchrony. SOC = second-order conditional sequences. SRT = serial reaction time. TC = tone counting task. VSC = visual stimuli counting.

### Why are the results so different?

Table 1. shows all of the studies that used a dual-task procedure to investigate implicit learning in artificial grammar learning and sequence learning paradigms that we have found in the literature. This review clearly shows that, at least for sequence learning, three groups of variables seem to interact with the presence of a secondary task, namely (a) variables that are related to the SRT procedure, (b) variables that are related to the type of secondary task, and (c) variables that are related to the connections between the main SRT task and the secondary task.

To begin with the SRT procedure, it appears that the structural complexity of the sequence mediates the impact that a secondary task has on learning the sequence. For instance, Cohen et al. (1990) provided evidence that sequences with at least some unique associations can be learned under attentional distraction, whereas ambiguous sequences require attention for learning. Similarly, deterministic sequence learning is more impaired by divided attention than probabilistic learning (Jiménez & Vázquez, 2005). Thus, learning more complex structures may depend more heavily on implicit and automatic learning processes. Hence, such more “implicit” SRT tasks should be less impaired by dual-tasking. This conclusion is supported by the fact that the degree of interference that results from dual-tasking decreases after extensive training on the SRT task (i.e., when an SRT task is automatized; see Cohen & Poldrack, 2008). The temporal characteristics of the SRT task could also interact with dual-tasking (e.g., Stadler, 1995). In fact, that variable could also be related to the connections between the main SRT task and the secondary task.

Let us now look in more detail at the second and most important group of variables that could influence the effects of a secondary task on SRT, namely the secondary task itself. Even if we assume that the attentional requirements of implicit learning are minimal, we can nonetheless expect to observe both a significant decrease in SRT performance under dual-task conditions, and a reduced transfer block effect when the training sequence is changed to another sequence or to random stimuli. Moreover, the magnitudes of these effects likely depend on the type of secondary task; therefore, comparing different secondary tasks should lead to different SRT results, which is precisely what Heuer and Schmidtke (1996) and Stadler (1995) observed. Surprisingly, however, those two papers are, to the best of our knowledge, the only ones that have directly addressed this problem. Furthermore, although both studies compared different secondary tasks, neither was specifically focused on exploring the differences in the attentional demands of each of the different secondary tasks that were used.

The aforementioned findings notwithstanding, we know from otherparadigms that different secondary tasks have different attentional requirements. For example, Roche et al. (2007) explored the relative demands of different secondary tasks that were performed during the learning block of a simple visual discrimination task. They observed that tone-counting (TC) could, under some conditions, be treated as a low-demand task, which is why it is often used in experiments that investigate age effects in sequence learning (e.g., see Experiment 3 of Frensch & Miner, 1994; Nejati, Garusi Farshi, Ashayeri, & Aghdasi, 2008). In fact, when we examine the attentional load experiments described in the sequence learning literature, TC was used as a secondary task in most of them (see Table 1.; see also Shanks, 2003, for a more detailed review of a few experiments with other secondary tasks). To the best of our knowledge, the effect of TC as a secondary task has only been contrasted once with the effect of another type of secondary task within a single experiment (Stadler, 1995). Interestingly, most of the artificial grammar learning experiments used RNG as a secondary task (see Table 1.), and never used TC in conjunction with this paradigm. Therefore, if we accept the general idea that implicit learning (as measured by both artificial grammar learning and sequence learning) requires at least a small amount of attentional resources, then the specific type of secondary task used in an experiment could easily bias the results for different reasons. Furthermore, TC does not seem to be the best secondary task to investigate attentional load effects.

Finally, specific parameters of a secondary task, such as the level of secondary task performance and the stimulus-onset asynchrony (SOA) may also influence sequence learning (see Frensch et al., 1994; Hsiao & Reber, 2001; Schumacher & Schwarb, 2009). Those parameters concern the third group of variables that are related to the connection between the secondary task and the main task. We will not discuss the details of those effects because they are not directly related to our research question, but it is worth noting that strong integration of the secondary task into the SRT task reduces dual-task interference (Rah, Reber, & Hsiao, 2000; Schmidtke & Heuer, 1997). It has also been shown that a secondary task can disturb learning by disorganizing SRT task consistency (e.g., by prolonging the SOA and thus disturbing the temporal organization of the sequence; cf. Schumacher & Schwarb, 2009; Stadler, 1995). Finally, Schumacher and Schwarb (2009) demonstrated that the degree of overlap between the processes involved in performing the secondary task and the SRT task (which was manipulated by means of task priority and SOA according to a psychological refractory period paradigm) modulates the way in which dual-tasking interferes with learning.

Although all of these approaches are interesting and should be taken into account, we believe that if one wants to measure the general attentional demands involved in sequence learning, one should first investigate the effects of attentional load using a highly demanding secondary task. Many results suggest that the RNG task fulfills this criterion (e.g., Brugger, 1997; Kareev, 1992; Rapoport & Budescu, 1997).

### The RNG task

RNG has been described as a good index of executive function because it requires high cognitive control (Baddeley, 1996; Kareev, 1992). When participants are asked to produce random sequences of digits, they must continuously control their behavior to prevent the occurrence of schematic responses (Van der Linden, Beerten, & Pesenti, 1998). Several studies have shown that people cannot react randomly and tend to deviate from randomness in numerous ways (i.e., the distribution of the possible options is usually unequal; participants tend to avoid repetitions and some type of counting is observed; for a review, see Towse & Neil, 1998). This tendency is particularly strong when participants are simultaneously engaged in other tasks (e.g., Miyake, Witzki, & Emerson, 2001).

Another important feature of the RNG task is that we can precisely assess the extent to which participants actually generate random numbers. On the basis of the assumption that it is more demanding to approach true random number generation than to merely generate regular series of numbers concurrently with performing the main task, a „randomness index” can be interpreted as a reflection of the extent to which attention is engaged by the task. In other words, because RNG uses cognitive resources, significant deviations from randomness under dual-task conditions indicate that these cognitive resources are directed toward performing the main task. Thus, measuring randomness continuously during the task allows one to better control participants’ engagement over time. Importantly, RNG is unaffected by repeated performance or practice (e.g., Jahanshahi, Saleem, Ho, Dirnberger, & Fuller, 2006). Therefore, RNG may be used during the entire SRT procedure without confounding practice effects.

### What type of knowledge is influenced by secondary task?

One of the main limitations of artificial grammar learning tasks and sequence learning tasks, especially of those that use deterministic sequences, is that participants tend to acquire some knowledge explicitly. Another issue that is still debated in sequence learning concerns the difficulty of determining whether performance on the SRT task reflects the amount of sequence knowledge that has been acquired, the amount of knowledge that is being expressed, or both (e.g., Frensch, Lin, & Buchner, 1998). We can avoid these problems by directly assessing participants’ knowledge of the sequence (or lack thereof) with a subsequent generation task. This task allows us to separate the learning and retrieval phases, and more importantly, to dissociate implicit and explicit knowledge components. In 2001, Destrebecqz and Cleeremans adapted this generation task in sequence learning by creating an inclusion condition, in which participants must generate previously viewed sequences, as opposed to an exclusion condition, in which they are required to inhibit the influence of prior knowledge by generating new sequences. They used the process dissociation procedure developed by Jacoby (1991) with the underlying assumption that automatic and controlled influences of memory may (under certain conditions) provide opposite results (Jacoby, 1998). Thus, generating the sequential regularities under inclusion condition should reflect both implicit and explicit learning, whereas the ability not to do so under exclusion condition should demonstrate the explicit character of acquired knowledge. Both influences can usually be observed in SRT results, which reflects the fact that the task is not process pure (i.e., the performance depends on both implicit and explicit knowledge). Depending on learning conditions, implicit and explicit processes could contribute differently to performance (see Destrebecqz & Cleeremans, 2001). We assume that a similar difference should be apparent when attention is diverted by a secondary task; that is, if indeed implicit learning is impaired in dual-task conditions, then we should observe impaired performance in the generation task. Importantly, the generation task gives us the opportunity to assess acquired knowledge independently from the indirect learning phase. This procedure makes it possible to have participants perform the SRT task under dual-task conditions and then to test dual-task interference in sequence learning in the generation task with no effect of the secondary task for the test itself.

In this paper, we investigated the detrimental effect of a secondary RNG task on sequence learning (Experiments 1a and 1b). In Experiment 2, the differences between the effects of two distinct secondary tasks were addressed by contrasting RNG with TC. In all three experiments, we evaluated participants’ knowledge of the sequence (or lack thereof) directly using a subsequent generation task.

## EXPERIMENT 1a

The aim of Experiment 1a was to investigate the role of a highly demanding secondary task on sequence learning. We asked participants in the experimental condition to perform RNG and the SRT task simultaneously. Participants were required to generate digits during all blocks of the SRT with no direct instruction about the required frequency of their responses. However, they were required to pay attention to both tasks equally. The general level of RNG randomness was measured throughout all of the SRT learning blocks (including the block in which the training sequence was transferred to another block) to assess their general attentional requirements. We assume that this highly demanding task will interfere with sequence learning, which will thereby result in disturbed patterns of performance in both the SRT and generation tasks.

### Method

#### Participants

A total of 40 undergraduate students in psychology from the Université Libre de Bruxelles (35 female, five male participants) voluntarily participated in the experiment in exchange for course credits. The average age of the participants was 20.3 years (range 18-24 years). Participants were randomly assigned to one of two experimental conditions, which were determined according to the attentional demands of the task. In the first condition (the control condition), participants simply performed the SRT task as a single task, whereas in the second condition (the RNG condition), they were required to perform a simultaneous secondary RNG Task.

#### Materials and Procedure

The experiment was run on a Macintosh Power PC 7600/132 computer. In the SRT task, participants were asked to react as fast as possible to stimuli that were presented on the computer screen. The stimulus could be presented at one of four positions marked by four dots arranged at 3 cm intervals along a horizontal line. Each screen position corresponded to one of four keys ([v], [b], [n], [m]) on a French AZERTY keyboard. The response-stimuli interval (RSI) was 250 ms long. The procedure consisted of a 60-trial training block followed by fifteen 96-trial experimental blocks with short breaks in between. Accuracy and RTs were recorded using the PsyScope software (Cohen, MacWhinney, Flatt, & Provost, 1993).

The order of stimulus presentation followed second-order conditional (SOC) sequences that determined the sequence of dot presentation (Reed & Johnson, 1994). Participants were trained with one of two possible SOC sequences (SOC 1: “3-2-4-1-3-4-2-3-1-2-1-4” or SOC 2: “3-2-3-4-1-2-4-3-1-4-2-1”) during Blocks 1-13 and were exposed to the other sequence in Block 14 (hence the transfer block). RTs typically increased during the transfer block, reflecting the fact that participants had become sensitive to the regularities of the training sequence. We label this specific increase in RT the *transfer block effect*. The training sequence was restored in Block 15. After completing all 15 blocks, the participants performed a subsequent generation task in which they were asked to reproduce the sequence of reactions from the SRT phase (inclusion condition) and then to try to avoid reproducing the sequential regularities (exclusion condition).

Throughout the SRT phase, participants in the RNG condition were asked to articulate a random digit between 0 and 9 aloud; the experimenter wrote down each digit. Instructions were identical to those in Dienes et al. (1991), except that we did not use a metronome to set the specific response timing to avoid a possible temporal interference with the primary SRT task. Afterwards, the randomness of the order of the digits from each participant was assessed for each block. We calculated two indexes of randomness: Redundancy and RNG. The redundancy index describes the distribution of possible responses (digits in the present study) in a RNG series (Towse & Neil, 1998). According to information theory assumptions (Shannon, 1948), a series of digits expresses a maximum amount of information when each possible option from the RNG set (each possible digit) is used with the same frequency. If the possible distribution of elements is not equal, redundancy in the material is observed. This is measured on a scale from 0 to100 for which a higher result indicates more redundant material. The RNG index reflects the distribution of pairs of elements (pairs of digits) in RNG series (Evans, 1978). Distribution of each possible pair of digits should be equal in perfectly random series. The results of the RNG index are expressed on a scale of 0-1, where 0 reflects an equal distribution of each possible pair of digits and 1 reflects the full predictability of the pairs. Henceforth, we will refer to this index as the *pair distribution index*. Detailed descriptions of both indexes can be found elsewhere (Barbasz, Stettner, Wierzcho, Piotrowski, & Barbasz, 2008; Towse & Neil, 1998). We calculated both of these indexes to ensure a more precise assessment of randomness. The redundancy index alone is not sensitive to the sequential regularities in the series of digits, unlike the pair distribution index (e.g., in the sequence consisting of *1*, *2* and *3*, the “1-2-3-1-2-3” sequence is fully random in terms of redundancy index but it is fully regular in terms of pair distribution).

### Results

#### SRT task

**Figure 1.**
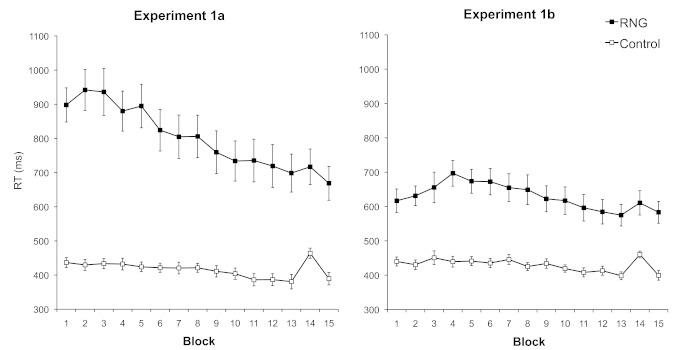
Mean reaction times (RTs) in the serial reaction time (SRT) task, plotted separately for the random number generation (RNG) and control conditions in Experiments 1a (left panel) and 1b (right panel). Error bars represent standard errors of the means.

Because the participants presented with either SOC 1 or SOC 2 in each condition were trained in the same manner, their RTs were combined for subsequent analyses. The overall learning effect was assessed using a two-way ANOVA with Block (the first 13 training blocks) as a within-subjects variable and Condition (RNG/control) as a between-subjects variable. As shown in Figure 1 (left panel), RTs decreased progressively during the task, and participants in the control condition reacted more quickly than participants in the RNG condition (mean RTs of 416 ms and 801 ms, respectively). This result is confirmed by significant main effects of block, *F*(12, 456) = 22.9, *MSE* = 106,097.05, *p* < .001, η^2^ = .38, and condition, *F*(1, 38) = 43.5, *MSE* = 22,247,585.59, *p* < .001, η^2^ .53. The Condition × SRT Block interaction was also significant, *F*(12, 456) = 10.1, *MSE* = 46,720.14, p < .001, η^2^ .21.

We now turn to the transfer block effect, measured by comparing the RTs from Block 14 (the transfer block) with the average of the RTs obtained in the adjacent regular blocks (Blocks 13 and 15) using a repeated measures analysis. This index provides a direct measure of sequential knowledge acquired during training. An ANOVA with Transfer (Block 14 vs. the average of Blocks 13 and 15) as a within-subjects variable and Condition (RNG/control) as a between-subjects variable revealed significant main effects of condition, *F*(1, 38) = 26.4, *MSE* = 1,520,752.34, *p* < .001, η^2^ .41, and transfer, *F*(1, 38) = 27.2, *MSE* = 62,875.05, *p* < .001, η^2^ .42. RTs increased by approximately 56 ms in Block 14. Moreover, the effects interacted; the increase in RTs in Block 14 was significantly larger in the control condition than in the RNG condition (79 ms vs. 34 ms), *F*(1, 38) = 4.4, *MSE* = 10,297.31, *p* = .041, η^2^ .10. However, despite a difference in magnitude, the transfer block effect was significant in both the control and the RNG conditions, *t*(19) = 4.95, *p* < .001, and *t*(19) = 2.31, *p* < .05, respectively.

#### RNG task

In this experiment, the participants seemed to thought they were required to generate a random digit for each and every key-press (the instruction did not state explicitly how often the random numbers should be generated). The participants generated an average of 81.5 digits for each block and approximately 96 digits during most of the blocks. This numbers corresponds to the 96 trials of the SRT task. To precisely assess the changes in the attentional requirements of SRT performance in the RNG condition, we calculated redundancy and pair distribution indices of randomness. The mean redundancy (distribution of possible elements) for the first 13 blocks of trials was quite low (3.38 out of 100), and the mean pair distribution for those blocks was 0.3. The indexes were analyzed using ANOVAs with Block as the within-subjects variable (Blocks 1 to 13). The block effect for the redundancy was significant, indicating that it slightly increased over time, *F*(12, 228) = 2.05, *MSE* = 5.99, *p* = .021, η^2^ .097. However, there was little redundancy in general and the increase was weak (from 2.8 in Block 1 to 3.8 in Block 13). In addition, pair distribution did not significantly differ as a function of training blocks (*F* < 1). A second set of ANOVAs was performed on both types of indexes in which Transfer was the within-subjects variable. Neither the redundancy index nor the pair distribution index for transfer block differed from that of adjacent blocks, *F*(1, 19) = 1.74, *MSE* = 2.22, *p* = .18, η^2^ .09, and *F* < 1 for the redundancy and pair distribution, respectively. In other words, the participants in the RNG condition produced slightly more redundant digits over time. Nonetheless, the overall redundancy remained low, and participants did not produce more regular pairs of elements with time. Moreover, the participants did not produce significantly less random material during the transfer block.

#### Generation task

To assess generation performance, we computed the number of generated chunks of three elements (triplets) that were part of the training sequence. A participant who possessed perfect knowledge of the sequence could produce a maximum of 94 training triplets because the generated sequences were 96 trials in length. Therefore, to obtain inclusion and exclusion scores for each subject, we divided the observed number of triplets that were part of the training sequence by the total number of produced triplets (94). Because we did not account for repetitions (participants had been instructed not to produce repetitions), the chance performance level was .33.

**Figure 2.**
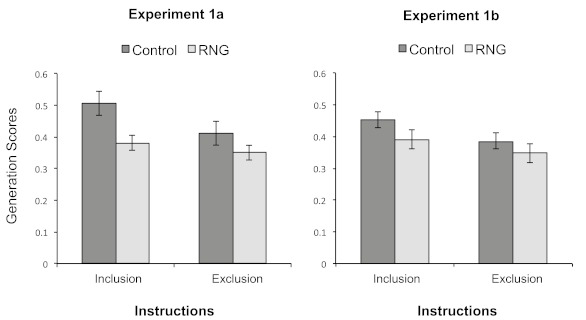
Mean proportions of generated second-order conditional transitions (SOCs) that were part of the training sequence (i.e., mean generation scores), for both conditions under inclusion or exclusion instructions in Experiments 1a (left panel) and 1b (right panel). Error bars represent standard errors of the means.

Figure 2 (left panel) shows average exclusion and inclusion scores for both conditions. It appears that more sequential elements were produced under inclusion than under exclusion instructions and that participants in the RNG condition generally produced fewer sequential triplets than participants in the control condition. This finding is confirmed by a two-way ANOVA with Instruction (exclusion/inclusion) as a within-subjects variable and Condition (Control/RNG) as a between-subjects variable in which both main effects were significant. The significant main effect of instruction type indicates that inclusion scores are higher than exclusion scores (.44 vs. .38), *F*(1, 38) = 6.8, *MSE* = 0.076, *p* < .05, η^2^ .15. The main effect of condition was also significant: Participants in the RNG condition produced fewer sequential triplets than participants in the control condition regardless of instruction type (.36 vs. .46), *F*(1, 38) = 5.9, *MSE* = 0.172, *p* = .020, η^2^ .13. In contrast to our expectations, the Instruction × Condition interaction was not significant, *F*(1, 38) = 1.8, *MSE* = 0.0202, *p* = .18, η^2^ .04. In other words, the pattern of performance in the generation task did not differ between our two groups of participants.

### Discussion

To summarize, we observed the classical effect of sequence learning characterized by both a significant decrease in RT during the task and a significant increase in RT during the transfer block. SRT performance was impaired under dual-task conditions, which concurs with reports in previous studies (Jiménez & Vázquez, 2005; Shanks et al., 2005; Shanks & Channon, 2002). We observed a general effect of secondary task performance: The average RTs were almost twice as long in the RNG condition than they were in the control condition. We also observed a significant interaction between Condition and Block, which revealed a steeper learning curve in the RNG condition. These results may suggest that in dual-task conditions, participants must learn to manage the RNG task performance to be able to perform the SRT task (i.e., the RNG task was a demanding one). An alternative explanation might be obtained by analyzing the pair distribution and redundancy indexes. The results of these index calculations suggest that the sequences of numbers generated in the RNG task were increasingly redundant (i.e., more regular) from block to block. This result can be interpreted as a change in task priority from prioritizing the RNG task (during the first blocks of the task) to prioritizing the SRT task. Finally, these results might not necessarily mean that participants’ performance was in any way impaired under dual-task conditions. It might be the case that the different learning slopes would stem from the very short RTs in the control group.

As expected, we observed a smaller transfer block effect under dual-task conditions. Importantly, the transfer block effect was significant in both conditions despite the difference in the magnitude of it. This effect could be related to theoretical proposals that, at least to some extent, SRT task performance depends on explicit learning (Curran & Keele, 1993; Jiménez & Méndez, 1999; Shanks, 2003). Based on this assumption, we could say that sequence learning occurs even under secondary task load because the secondary task may influence implicit sequence learning, explicit sequence learning, or both. To differentiate between the contributions of the two types of knowledge acquired under RNG or control conditions, the results of generation task were computed. However, performing a RNG secondary task during the learning phase did not specifically interfere with performance on any of the generation subtasks. Performance in both exclusion and inclusion task conditions was impaired under dual-task conditions, which suggests that the influence of RNG is not specific to any type of knowledge. Instead, it results in a general overload of the attentional system. To replicate the findings of this first study, we conducted Experiment 1b with a few small changes to the procedure.

## EXPERIMENT 1b

The aim of Experiment 1b was to replicate the findings of Experiment 1a. Because general overload of the attentional system could explain the results obtained in Experiment 1a, we decided to use a less demanding version of the secondary task (participants generated a digit on every fourth key-press during the SRT task).^1^ We also measured a baseline RNG performance level (i.e., performance on the RNG task as single task) to assess the level of participants’ engagement in performing RNG as the secondary task. We asked participants in the experimental condition to perform the RNG and SRT tasks simultaneously. As in Experiment 1a, we expected that performing a secondary RNG task in conjunction with an SRT task should lead to slower RTs and reduced transfer block effect size. The contributions of the explicit and implicit influences of memory to performance on the generation task should also differ under dual-task conditions. Finally, if SRT task performance engages participants’ attention, the level of RNG randomness should differ from the baseline level.

### Method

#### Participants

A total of 38 undergraduate students at the Université Libre de Bruxelles (eight males and 30 females, none of whom had participated in Experiment 1a) took part in this experiment in exchange for course credits. The average age of the participants was 19.8 years (range 17-33 years). Participants were randomly assigned to the control condition (*n* = 20) or to the RNG condition (*n* = 18).

#### Materials and Procedure

In Experiment 1b, we used the same tasks and procedures as in Experiment 1a, with two exceptions. Participants in the RNG condition were asked to articulate a random digit between 0 and 9 aloud after each fourth key-press during the SRT task (unlike in Experiment 1a, where they spontaneously generated digits after each reaction). This instruction required participants to utter a digit after every fourth key-press; thus, the participants needed to count when the response should be made. In addition, to assess the baseline level of RNG performance, participants were asked to generate random digits as a single task before the SRT task. To complete this task, the participants were asked to articulate random digits between 0 and 9 aloud. The task lasted approximately 3 min, and approximately 60 responses were recorded from each participant.

### Results

#### SRT task

The data presented in the right panel of Figure 1 were analyzed in the same manner as in Experiment 1a. An ANOVA with Block (the first 13 training blocks) as within-subjects variable and Condition (RNG/control) as a between-subjects variable revealed a main effect of block: RTs decreased over time, *F*(12, 432) = 10.6, *MSE* = 23,708.56, *p* < .001, η^2^ .23. The main effect of condition was also significant, *F*(1, 36) = 33.2, *MSE* = 5,175,847.33, *p* < .001, η^2^ .48. As in Experiment 1a, participants in the RNG condition reacted more slowly than controls (634 ms vs. 429 ms). The difference between the mean RTs from Blocks 1 and 13 was nearly identical in both conditions (42 msand 41 ms), which suggests that both groups had similar learning effects, but the Block × Condition interaction was significant, *F*(12, 432) = 2.9, *MSE* = 6,491.67, *p* = .001, η^2^ .075. This result might be because the RTs of participants in the RNG condition increased monotonically from Block 1 to Block 4, and they only began to decrease regularly (monotonically) in Block 4. The difference in RTs between Blocks 4 and 13 was much larger for participants in the RNG condition than in the control condition (122 ms vs. 41 ms). For that reason, we repeated two-way ANOVA (Block × Condition) for Blocks 4 to 13 only. The main effects of both block and condition remained significant, *F*(9, 324) = 18.2, *MSE* = 29,508.73, *p* < .001, η^2^ .33, and *F*(1, 36) = 32.6,*MSE* = 4,108,958.68, p < .001, η^2^ .47, respectively. Most importantly, the Block × Condition interaction also remained significant, *F*(9, 324) = 4.7, *MSE* = 7,696.51, *p* = .001, η^2^ .116. This result confirms the impression from Figure 1 (right panel) that the slope of the learning curve in the RNG group is steeper than that of the control group. To get a better idea of the impact of dividing attention on the acquisition of sequential regularities, we looked at the transfer block effect; that is, we examined the difference in RTs when the training sequence was replaced with a different one at Block 14.

As in Experiment 1a, ANOVA with Transfer (Block 14 vs. the ave-rage of Blocks 13 and 15) as a within-subjects variable and Condition (RNG/control) as a between-subjects variable revealed the main effect of condition, *F*(1, 36) = 25.0, *MSE* = 517,633.11, p < .001, η^2^ .41. The transfer block effect was also significant, *F*(1, 36) = 38.8, *MSE* = 41,094.99, p < .001, η^2^ .52, and there was an average increase in RTs of 47 ms when a new sequence was suddenly presented. Although this transfer cost was almost twice as large for participants in the control condition as it was for participants in the RNG condition (62 ms and 32 ms, respectively), the interaction between Transfer and Condition was only marginally significant, *F*(1, 36) = 3.9, *MSE* = 4,160.21, *p* = .055, η^2^ .098. As in Experiment 1a, separate t tests revealed significant transfer block effects in both the control, *t*(19) = 6.2, *p* < .001, and the RNG conditions, *t*(17) = 2.8, *p* = .013.

#### RNG task

As in Experiment 1a, we calculated redundancy and pair distribution indexes of randomness. Means for the first 13 blocks were 7.07 out of 100 for the redundancy index and 0.16 for the pair distribution index. Two separate ANOVAs (one for each index) with Block as the within-subjects variable (Blocks 1-13) were performed and showed that the main effect of block was not significant for either the redundancy index (*F* < 1.6) or the pair distribution index (*F* < 1). A second set of ANOVAs was conducted for both indexes, with Transfer as the within-subjects variable. The main effect of transfer was also not significant (for either redundancy or pair distribution, *Fs* < 1). The average baseline redundancy equaled 4.22 and was significantly less redundant than in Blocks 1-13, *t*(19) = 4.54, *p* < .001. Contrasting results were observed when considering the pair distribution index; the baseline pair distribution equaled .25 and was significantly higher (less random) than the pair distribution index during the SRT task performance, *t*(19) = -7.64, *p* < .001.

#### Generation task

Generation performance under inclusion and exclusion instructions is presented in Figure 2 (right panel). An ANOVA with Instruction (inclusion/exclusion) as a within-subjects variable and Condition (control/RNG) as a between-subjects variable revealed a main effect of instruction: the sequence reproduction scores were significantly higher under inclusion instructions than under exclusion instructions (.42 vs. .36), *F*(1, 34) = 5.5, *MSE* = 0.055, *p* = .025, η^2^ .14. However, neither the main effect of condition, *F*(1, 34) = 2.6, *MSE* = 0.44, *p* = .11, η^2^ .07, nor the Instruction × Condition interaction (*F* < 1) was significant.

### Discussion

To summarize the results of Experiment 1b, we observed both a significant effect of learning as well as a significant transfer block effect, which were consistent with typical sequence learning findings. SRT task performance was impaired under dual-task conditions. It is worth noting that the mean RTs for participants in the RNG condition were approximately 200 ms faster than in Experiment 1a. This finding seems to confirm that this new RNG procedure is less demanding. The redundancy and pair distribution index data also support that conclusion: In this experiment, the randomness of RNG task performance was comparable throughout all of the blocks. We therefore conclude that this version of the RNG task is more appropriate than the one used in Experiment 1a.

A significant interaction between Condition and Block was observed again, but the overall pattern of results looks different in this experiment. The results suggest that under dual-task conditions, RT increases up to the Block 4, after which the classical decrease in RT is observed. In our opinion, this result suggests that even this easier version of the RNG task was still quite demanding for participants.^2^ During the first four blocks of the main SRT task, the participants still had to learn how to manage the secondary RNG task so that they would be able to perform them simultaneously later. Randomness indexes partially confirm this hypothesis: The pair distribution index reveals that distribution of pairs under dual-task conditions is more regular than the baseline distribution. However, the redundancy index results show the opposite.

As in the previous experiment, a transfer block effect was observed in both conditions, but its amplitude was smaller under dual-task conditions. To interpret this transfer block effect, the results of a sequence generation task were computed. In this experiment, similar performance in both the RNG and control conditions indicated that dual-tasking during the training phase did not result in an overall decrease in the proportion of sequential fragments reported in the subsequent generation task. Interestingly, inclusion scores were much higher than exclusion scores; this finding suggests that the level of involvement of implicit knowledge was low. This result could be interpreted as evidence that a strong explicit knowledge component is acquired during the SRT task, but the results of the secondary task performance do not support this explanation. An alternative explanation, of course, is that both conditions developed an explicit learning component that could not be expressed in the RNG condition because resources were involved with secondary task execution. Thus, it is more likely that performing the secondary task interfered with the procedural learning of motor reaction, that is, the observed result is more likely to be the result of a time scale disorganization (see Stadler, 1995) than of impaired knowledge representation.

## EXPERIMENT 2

In the final experiment, we went one step further in our attempt to address the question of the way in which attentional load influences sequence learning, and compared two types of secondary tasks. Thus, one group of participants performed an RNG task during the SRT task, as in Experiments 1a and 1b. Another group of participants was presented with the most popular secondary task used in previous studies: TC; and there was also a control condition. We used the less demanding version of the secondary RNG task as we did in Experiment 1b (participants had to articulate a digit every fourth key-press in SRT). We also measured the baseline performance in the RNG task. To assess the randomness generation ability of participants more correctly, this baseline was recorded during one block of an SRT-like task with no reaction demand. As in previous studies, we predicted impaired performance among participants in the RNG group as measured by overall reaction time, transfer block effect size, generation task performance and the degree of RNG randomness.

### Method

#### Participants

A total of 60 students from Jagiellonian University (20 male and 40 female) voluntarily took part in Experiment 2 in exchange for course credits. The average age was 20.4 (range 19-25). Participants were randomly assigned to the control (*n* = 18), RNG (*n* = 18), and TC (*n* = 24)conditions.

#### Materials and Procedure

In Experiment 2, we added a TC condition to compare the specific influence of TC versus RNG on SRT task performance. As we mentioned in the general introduction, TC is commonly used in the literature as a secondary task during sequence learning (e.g., Jiménez & Vázquez, 2005). In this condition, we presented a low-pitched (1000 Hz) or high-pitched (2000 Hz) tone for 50 ms through headphones on 25% of the trials (to make the degree of TC disturbance more comparable to that of RNG). In total, 24 tones were presented randomly during each block, including an average of eight to 16 target tones. The participants were asked to keep a count of the number of high-pitched tones while simultaneously proceeding with the SRT task, and they were asked to report this number at the end of each block.

We also slightly changed the method of measuring the RNG baseline. To make the RNG baseline more relevant to the SRT task situation, we asked participants to look at stimuli (dots) that were presented in the same way as in the SRT task and articulate a random digit between 0 and 9 aloud after every fourth stimulus. However, unlike the real SRT task situation, participants were not required to react to the visual stimuli with key-presses.

We also counterbalanced the order of the two subtasks in the generation task in this final experiment. Thus, half of the participants performed the inclusion task first, followed by the exclusion task, and the second half performed generation tasks in the reverse order.

All other materials and procedures were the same as those used in Experiment 1b.

### Results

#### SRT task

**Figure 3.**
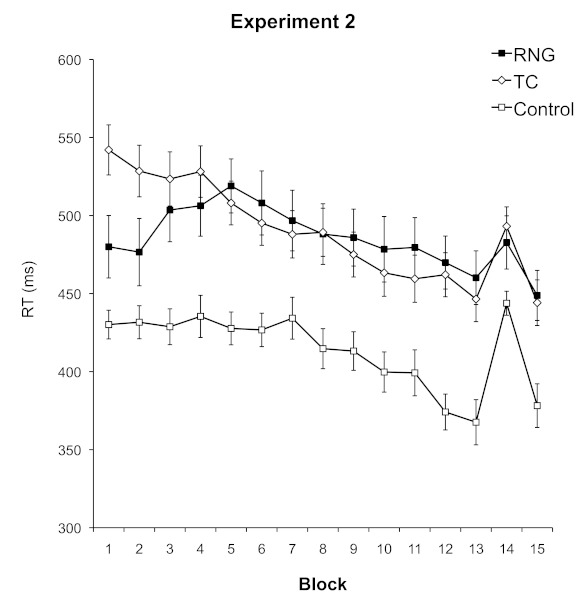
Mean reaction times (RTs) in the serial reaction time (SRT) task (Experiment 2), plotted separately for the control, random number generation (RNG), and tone counting (TC) conditions. Error bars represent standard errors of the means.

As in previous experiments, the ANOVA with Block (the first 13 training blocks) as a within-subjects variable and Condition as a between-subjects variable was performed; however, in this experiment, three different conditions were used: Control, RNG, and TC. The analysis showed a significant main effect of block: The differences between RTs in the first and 13th blocks was 63 ms, *F*(12, 684) = 29.2, *MSE* = 318,605.740, *p* < .001, η^2^ .34. The main effect of condition was also significant, *F*(2, 57) = 7.8, *MSE* = 487,482.33, *p* = .001, η^2^ .21.RTs in the control condition (414 ms) were significantly faster than RTs in the RNG (488 ms), *F*(1, 34) = 12.8, *MSE* = 650,313.07, *p* = .001, η^2^ .27, and in the TC (493 ms), *F*(1, 40) = 14.6, *MSE* = 832,844.79, p < .001, η^2^ .26, conditions, but there was no significant difference in RTs between the latter two conditions (t < 1). The Block × Condition interaction was also significant, *F*(24, 684) = 4.7, *MSE* = 4,262.73, *p* < .001, η^2^ .14. As shown in Figure 3, the RTs of participants in the RNG condition increased until Block 5 and then began to decrease according to the same pattern as the RTs of participants in the control and TC conditions. This result suggests that the non-monotonic effect of learning that was previously observed in Experiment 1b was not accidental. Thus, we ran an additional ANOVA (Block × Condition) with only Blocks 5 to 13. This ANOVA yielded significant effects of block, *F*(8, 456) = 34.6, *MSE* = 23,517.17, *p* < .001, η^2^ .37, and Condition, *F*(2, 57) = 7.8, *MSE* = 321,834.41, *p* = .001, η^2^ .21. However, these two factors did not interact as they did in Experiment 1b, *F*(16, 456) = 1.3, *MSE* = 885.89, *p* = .19, η^2^ .04.

As in previous experiments, the ANOVA with Transfer as a within-subjects variable and Condition as a between-subjects variable revealed a main effect of transfer. RTs increased by an average of 49 ms when the training sequence was replaced by a different sequence in Block 14, *F*(1, 57) = 116.2, *MSE* = 70,713.68, *p* < .001, η^2^ .67. The significant Transfer × Condition interaction, *F*(2, 57) = 6.7, *MSE* = 4,101.097, *p* = .002, η^2^ .19, indicates that there were systematic differences among the three conditions. In fact, RTs during the transfer block increased by 71, 48, and 29 ms in the control, TC, and RNG conditions, respectively. To compare the transfer block effect between the two dual-task conditions, we repeated the Transfer × Condition ANOVA but excluded the control condition. The main effect of transfer remained significant, *F*(1, 40) = 55.2, *MSE* = 29,774.02, *p* < .001, η^2^ .58. More importantly, however, there was also a marginally significant Transfer × Condition interaction, *F*(1, 40) = 3.6, *MSE* = 1,964.99, *p* = .063, η^2^ .084, which confirms that there was a larger transfer block effect in the TC condition than in the RNG condition. Nevertheless, the transfer block effect in both conditions was significant, *t*(17) = 3.8, *p* = .001, and *t*(23) = 6.9, *p* < .001, for the TC and RNG conditions, respectively. These analyses suggest that generating numbers at random during the SRT task may be more detrimental than counting high-pitched tones to sequence learning.

#### RNG task

The average index of redundancy for Blocks 1-13 was 10.2 out of 100 and the index of pair distribution was .17. As in Experiment 1b, separate ANOVAs for randomness with Block as the within-subjects variable (Blocks 1-13) showed no effects of block for either redundancy or pair distribution (*Fs* < 1). A second set of ANOVAs was conducted for both indexes using Transfer as the within-subjects variable. The transfer block effects were also not significant (*Fs* < 1.5). The average baseline redundancy equaled 6.18 out of 100 and was significantly less redundant than the redundancy in Blocks 1-13, *t*(17) = -6.66, *p* < .001. The results for the pair distribution index were not significant: The baseline performance equaled .2 and was comparable to the pair distribution index that was observed during SRT performance, *t*(17) = 1.29, *ns*.

#### Generation task

**Figure 4.**
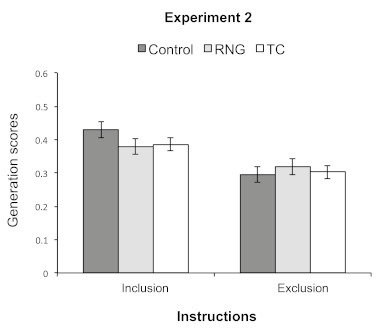
Mean proportions of generated second-order conditional transitions (SOCs) that were part of the training sequence (i.e., mean generation scores) under inclusion or exclusion instructions in Experiment 2. Error bars represent standard errors of the means.

Generation scores are presented in Figure 4. The ANOVA with Instruction (inclusion/exclusion) as a within-subjects variable and Condition (RNG/TC/control) as a between-subjects variable yielded a significant main effect of instruction, *F*(1, 57) = 30.9, *MSE* = 0.251, *p* < .001,η^2^ .35; more sequential elements were reproduced in the inclusion (.40) condition than in the exclusion condition (.30).^3^ However, the Condition factor was not significant, *F* < 1, nor was the Instruction × Condition interaction, *F*(2, 57) = 1.5, *MSE* = 0.013, *p* = .22, η^2^ .055.

### Discussion

In the second experiment, we again observed the presence of sequence learning, as evidenced by a progressive decrease in RTs as a function of training and a specific increase in RTs during the transfer block. Sequence learning was impaired when participants were asked to perform either secondary task: There was no difference in impairment between TC and RNG secondary tasks. The pattern of results was similar to that in Experiment 1b. The results of randomness index calculations partially confirm that the simultaneous performance of RNG and SRT tasks was indeed attentionally demanding. The redundancy index revealed more redundant performance under dual-task conditions. However, pair distribution index results were not significant (i.e., they do not support a conclusion that dual-task conditions were more difficult than control conditions).

As in previous experiments, the transfer block effect was significant in all conditions, but its magnitude was reduced under dual-task conditions. Interestingly, there was also a difference between the two types of secondary tasks, suggesting that an RNG task is more demanding than a TC task and that it impairs SRT performance to a larger extent. To qualify these results, the generation task data were calculated. As in Experiment 1b, no effects of secondary task performance were observed in either the inclusion or the exclusion conditions. Inclusion scores were again much higher than exclusion scores (in this experiment, the exclusion scores were even below the chance performance level), suggesting that there was little involvement of implicit knowledge.

## GENERAL DISCUSSION

The main empirical goal of the present paper was to explore the extent to which RNG could be used as a secondary task in a SRT paradigm. As expected, we observed significant learning and transfer block effects in all experiments that confirm that participants acquire knowledge about the sequential regularities of the material. Importantly, two sets of data indicate that sequence learning was systematically impaired when an RNG task was used as a secondary task. First, simultaneous performance of RNG and SRT tasks prolongs RTs by approximately 200 to 400 ms, depending on the task version employed. Second, although we observed significant transfer block effects in all groups, their magnitudes were smaller under dual-task conditions. Interestingly, in addition to the general dual-task interference, Experiment 2 revealed that the transfer block effect was larger when participants had to perform TC as a secondary task than when they had to perform RNG. This result confirms that RNG is a more demanding task than TC. This difference notwithstanding, our results also show that participants remain capable of learning and expressing sequence knowledge even under dual-task conditions. This finding raises questions about what type of knowledge is measured by the transfer block effect, which is an issue we will return to in the next section.

Other data from a subsequent sequence generation task in which implicit and explicit processes can be isolated did not clearly indicate the role that an RNG secondary task plays in the expression of sequential knowledge. Indeed, the expected interaction between Instructions and Conditions was never observed, which leads us to conclude that the secondary task did not interfere exclusively with either implicit or explicit knowledge. However, the main effect of Condition that we observed in the first two experiments reflects the influence of the RNG task on sequence generation task performance. Finally, there was also a main effect of Instructions in all experiments (i.e., inclusion scores were much higher than exclusion scores). This finding is interesting, because it suggests that implicit knowledge was barely involved in the present experiments (or even no implicit knowledge at all in the case of Experiment 2, where exclusion scores were marginally lower than chance). We will return to this point in the next section.

Finally, the randomness indexes (redundancy and pair distribution) allow for a more precise assessment of the secondary task performance. The averages of both redundancy and pair distribution were relatively low in general, that is, even when RNG was performed simultaneously with the SRT task, participants were able to generate non-redundant sequences with equally distributed choices. This result suggests that the SRT task is not a very demanding one when conducted using spatial (and compatible) stimuli. Importantly, redundancy index data indicate that RNG was less random when it was used as a secondary task than it was at baseline; pair distribution results were inconclusive. This finding suggests that participants do indeed engage their attention in the SRT task performance to some extent. It is also possible, however, that participants simply bear the burden of simultaneous execution of two tasks. We also compared the level of participants’ engagement in the RNG task over time to detect any effect of practice. No such effect was observed, which makes RNG a good secondary task candidate (see Jahanshahi et al., 2006).

In summary, our results confirm that RNG impairs SRT task performance and thus that it can be successfully used as secondary task in sequence learning studies. More importantly, our results suggest that, at least in terms of the transfer block effect, RNG is more demanding than TC. However, an interesting question remains unanswered: What type of learning is impaired by RNG in the SRT task? To answer this question we should take a closer look into the transfer block effect and into the sequence generation task results.

### Transfer block effect and generation task

Classically, the transfer block effect in sequence learning has been interpreted as the manifestation of implicit sequence learning (see Nissen & Bullemer, 1987; Shanks & Channon, 2002) or as a test of implicit knowledge expression (Frensch, Lin, & Buchner, 1998). Because we have not controlled knowledge expression, we will focus on implicit sequence learning. Many studies suggest that knowledge acquired during an SRT task is not process pure (i.e., that it is at least partially explicit; e.g., Destrebecqz & Cleeremans, 2001). In this context, it seems probable that the transfer block effect reflects the influence of both implicit and explicit components of knowledge acquired over the course of the SRT task or possibly just the procedural learning of motor reaction (e.g., intentional stimulus-response translations [see Hommel, 2000] that make it necessary to react even when the stimuli are randomly presented). This possibility seems to be supported by the Woltz et al. (2000) study that suggests that the transfer block effect in sequence learning might result not only from implicit knowledge acquisition but also from procedural learning of motor reactions. However, if we agree that the transfer block effect does not necessarily reflect implicit sequence learning, then the results of a secondary task’s influence on SRT task performance should be reinterpreted.

Our data demonstrate that participants in all three RNG conditions responded much more slowly than participants in the control conditions. Indeed, participants in the former (RNG) group required nearly twice the amount of time to complete the SRT task in Experiment 1a that control participants needed. With our alternative explanation, the higher overall RT during learning might reflect either the interference of secondary task performance with the expression of any knowledge or the cost of attentional overload, which is not specific to implicit sequence learning. Furthermore, we could interpret the transfer block data analogously. There is a reduced transfer block effect in the RNG condition, but it is important to note that the transfer block effect was nonetheless observed even under dual-task conditions. If we assume that this sudden increase in RTs does not reflect implicit learning alone, it is possible that other components of learning observed in the SRT task are also influenced by the performance of a secondary task. It should be noted that participants in our studies also performed RNG during the transfer phase. In this context, the significant transfer block effect in the RNG condition may suggest that participants still acquired some implicit knowledge about the sequence structure but that the secondary task influenced either the explicit knowledge expression or the procedural learning of motor reactions (Frensch et al., 1998; Jiménez & Vázquez, 2005).

In summary, several explanations can be put forward to interpret the reduced transfer block effect that is observed under dual-task conditions: (a) impaired implicit sequence learning (as proposed by Jiménez & Vázquez, 2005; Shanks et al., 2005; Shanks & Channon, 2002),(b) impaired knowledge expression, (c) impaired explicit sequence learning, and/or (d) impaired procedural learning of motor reactions. It is impossible to dissociate the ways in which the secondary task influences all of the observed aspects of learning using an SRT task alone, so other measures of separate components of learning should be used to address this question. It is therefore worth discussing the results of our generation task results in this context.

We administered a sequence generation task to assess the ways in which the secondary task influences implicit and explicit learning separately. However, as reported above, we have not observed any specific influence of RNG (i.e., neither inclusion nor exclusion scores were exclusively impaired by the secondary task performance during SRT); we only observed a general impairment in generation task performance (Experiments 1a and 1b). These results suggest that the secondary task did not affect implicit sequence learning, but they also fail to confirm that RNG influences explicit knowledge. Taken together, these observations may indicate that the secondary task influences the procedural learning of motor reaction, or that the implicit component of learning was so minimal that an interaction could not be observed due to a floor effect.

In conclusion, we argue that the transfer block effect could actually reflect the combined influence of implicit, explicit, and motor components of learning. A few authors have already proposed such an interpretation (e.g., Woltz et al., 2000), but in our opinion, the transfer block effect should also be used to discuss the effects of a secondary task on sequence learning. Following this interpretation, we cannot draw conclusions about which type of knowledge is actually affected by attentional load if we rely solely on data about transfer block effects. Our generation task results confirm this interpretation to some extent: They suggest that the influence of RNG and TC on SRT task performance may not be due to implicit knowledge impairment simply because the implicit component of knowledge was very small in all of our studies. In other words, we propose that performing a secondary task disturbs sequence learning, but not necessarily the implicit component of it. This proposal should be investigated in future studies. Furthermore, we list other interesting research questions and methodological problems that could be addressed in future studies investigating RNG influence on SRT task performance below.

### RNG: Future study directions

In the final section of this article, we will focus on additional analyses and manipulations that should be investigated in future studies of influence of RNG on SRT task performance, and we discuss some methodological weaknesses in our experiments upon which there is room for further improvement.

First, it seems obvious from analyzing randomness data that one of the advantages of RNG is related to the additional in-depth analysis that can be performed using randomness indexes. We have already presented some analyses in which we assessed the attentional demands of RNG and SRT task performance. However, additional in-depth analyses of SRT task performance accompanied by RNG could be imagined. For example, one may use both the redundancy and pair distribution indexes to correlate the level of engagement in RNG with SRT performance (by computing correlations between RNG performance and performance on SRT and generation tasks). It is also possible to analyze both individual differences in RNG (by comparing participants who are able to generate more or fewer random digits) and the impact of those differences on SRT learning effects. We have attempted to analyze data in this way, but we did not observe any significant results, most likely due to both the low variability of the RNG indexes and an insufficient number of participants in each experiment (at least for the individual difference comparison). However, investigation of these effects presents interesting possibilities for future studies.

Another interesting problem to investigate in future studies is the problem of RNG and SRT task synchronization. In this context, the frequency of random number generation during an SRT task should be discussed. Our results indicate that the version of an RNG task that was used in Experiment 1a was more demanding than the one used in Experiments 1b and 2, and it was most likely too difficult for participants. Accordingly, RNG should be used only on some SRT trials in future research. However, our second version of the RNG task (the “every fourth reaction” version) revealed some problems as well and should be improved further. In particular, it seems that in this condition, participants must actually perform three tasks (viz., performing SRT, RNG, and counting every fourth reaction). Altogether, it seems that internally triggered RNG is very difficult to control and the results are even more difficult to interpret. It should also be noted that with regular, external control of the generation of the digits, participants could also employ strategies to avoid attentional costs related to RNG task performance. For these reasons, we propose that the pace of the RNG task should be externally controlled in future studies. The requirement of random number generation may be indicated with a cue (e.g., a red dot) that would be integrated within the SRT task (i.e., the cue will be a part of the SRT stimulus).

Finally, some procedural suggestions for future studies utilizing RNG in an SRT paradigm should also be made. Most importantly, regardless of the RNG version, assessing the task synchronization between the SRT and RNG tasks would require data to be collected on a trial-by-trial basis. In all experiments, we collected the RNG data in a way that did not allow us to monitor this type of synchronization (we have not monitored the specific trial in the SRT task on which each random digit was generated; participants were instructed to generate digits and their responses were written down by the experimenter). If the synchronization between tasks was controlled (e.g., by means of a voice onset detection device), it would also be possible to compare performance on the main SRT task trials in which the RNG task is performed simultaneously (in our case it was every fourth key-press) with performance on control trials during which no digit is generated (all other trials). Finally, it would be plausible to search for, and to control if necessary, other variables that could influence performance on both the RNG and SRT tasks, such as the overlap between spatial and numeral representations. The SNARC effect could be one example (e.g., Dehaene, Bossini, & Giraux, 1993): It is possible that SRT reactions that are mapped to the left side of the screen are related to the generation of lower digits in RNG and that reactions mapped to the right part of the screen are related to higher digits in RNG.^4^ We also observed a non-monotonic function of SRT task performance when the SRT and RNG tasks were performed simultaneously (Experiments 1b and 2). These results suggest that ability to perform both tasks simultaneously requires learning. Therefore, it seems that a longer training phase in which participants learn how to perform the RNG and SRT tasks simultaneously should be utilized in future studies. We also did not control the task prioritization in our studies. We do not know whether participants pay more attention to the RNG task or to the SRT task, or whether they switch between the two tasks. This type of effect could also be controlled by task priority manipulation, which seems to have an impact on SRT task performance (see Schumacher & Schwarb, 2009). Finally, it would also be interesting to investigate our interpretation of the transfer block effect in the context of the acquisition and expression of implicit knowledge (Frensch et al., 1998). Thus, in future studies RNG should be used exclusively during acquisition and/or transfer blocks.

### Closing remarks

In conclusion, although it appears that RNG influences SRT task performance, it does not necessarily influence implicit sequence learning per se. In light of this conclusion, the consequences of the influence that a secondary task has on implicit knowledge need to be explored further. In this context, our studies offer at least one clear conclusion: RNG is a promising task that makes it possible to control the use of attentional resources during an SRT task.

## Footnotes

1 We assumed that this version of the RNG task would be less demanding for participants because it does not continually interfere with their performance (on every trial). In fact, participants could switch between SRT and RNG tasks with this version of the task.

2 The entire experimental situation was also more complex in the sense that participants needed to count to generate a response every fourth key-press. Participants may have needed these first four blocks to become accustomed to the experimental situation.

3 In all experiments we also assessed performance under inclusion and exclusion instructions by comparing generation scores to the chance performance level (.33). In Experiments 1a and 1b, generation scores in both instruction conditions were always above the chance level (*ps* < .05). Interestingly, this was not the case in Experiment 2. In this experiment, participants generated more sequential elements than chance only in the inclusion condition, *t*(59) = 4.0, *p* < .001; exclusion scores were not significantly different from the chance performance level, although they were marginally lower than it, *t*(59) = - 1.9, *p* = .06.

4 We thank Robert Gaschler for suggesting the investigation of the SNARC effect in context of sequence learning.

## ACKNOWLEDGEMENTS

This research was partially supported by a grant from the Polish Ministry of Science and Higher Education (N106 254837). In the course of preparation of this manuscript, Micha Wierzcho was also supported by the Support for Scientists’ International Mobility Program of Polish Ministry of Science and Higher Education. The authors would like to thank Anna Marzecová for her valuable comments on previous versions of this manuscript.
